# A Smartphone-Based Self-Management Intervention for Individuals with Bipolar Disorder (LiveWell): Qualitative Study on User Experiences of the Behavior Change Process

**DOI:** 10.2196/32306

**Published:** 2021-11-22

**Authors:** Geneva K Jonathan, Cynthia A Dopke, Tania Michaels, Clair R Martin, Chloe Ryan, Alyssa McBride, Pamela Babington, Evan H Goulding

**Affiliations:** 1 Department of Psychiatry and Behavioral Sciences Feinberg School of Medicine Northwestern University Chicago, IL United States; 2 Department of Pediatrics Loma Linda Children's Hospital Loma Linda, CA United States; 3 Department of Social Work UPMC Western Psychiatric Hospital Pittsburgh, PA United States; 4 Feinberg School of Medicine Northwestern University Chicago, IL United States

**Keywords:** behavioral intervention technology, mHealth, bipolar disorder, depression, illness management, smartphone, behavior change, early warning signs, self-management, qualitative, behavior, intervention, management, user experience, perception, utilization

## Abstract

**Background:**

Bipolar disorder is a severe mental illness characterized by recurrent episodes of depressed, elevated, and mixed mood states. The addition of psychotherapy to pharmacological management can decrease symptoms, lower relapse rates, and improve quality of life; however, access to psychotherapy is limited. Mental health technologies such as smartphone apps are being studied as a means to increase access to and enhance the effectiveness of adjunctive psychotherapies for bipolar disorder. Individuals with bipolar disorder find this intervention format acceptable, but our understanding of how people utilize and integrate these tools into their behavior change and maintenance processes remains limited.

**Objective:**

The objective of this study was to explore how individuals with bipolar disorder perceive and utilize a smartphone intervention for health behavior change and maintenance.

**Methods:**

Individuals with bipolar disorder were recruited via flyers placed at university-affiliated and private outpatient mental health practices to participate in a pilot study of LiveWell, a smartphone-based self-management intervention. At the end of the study, all participants completed in-depth qualitative exit interviews. The behavior change framework developed to organize the intervention design was used to deductively code behavioral targets and determinants involved in target engagement. Inductive coding was used to identify themes not captured by this framework.

**Results:**

In terms of behavioral targets, participants emphasized the importance of managing mood episode–related signs and symptoms. They also discussed the importance of maintaining regular routines, sleep duration, and medication adherence. Participants emphasized that receiving support from a coach as well as seeking and receiving assistance from family, friends, and providers were important for managing behavioral targets and staying well. In terms of determinants, participants stressed the important role of monitoring for their behavior change and maintenance efforts. Monitoring facilitated self-awareness and reflection, which was considered valuable for staying well. Some participants also felt that the intervention facilitated learning information necessary for managing bipolar disorder but others felt that the information provided was too basic.

**Conclusions:**

In addition to addressing acceptability, satisfaction, and engagement, a person-based design of mental health technologies can be used to understand how people experience the impact of these technologies on their behavior change and maintenance efforts. This understanding may then be used to guide ongoing intervention development. The participants’ perceptions aligned with the intervention’s primary behavioral targets and use of a monitoring tool as a core intervention feature. Participant feedback further indicates that developing additional content and tools to address building and engaging social support may be an important avenue for improving LiveWell. A comprehensive behavior change framework to understand participant perceptions of their behavior change and maintenance efforts may help facilitate ongoing intervention development.

## Introduction

Bipolar disorder (BD) is a severe mental illness characterized by recurrent episodes of depressed, elevated, and mixed mood states [1]. Episode recurrence, prolonged episodes, and interepisode symptoms often adversely impact psychosocial functioning and quality of life [2-7]. The addition of psychotherapy to pharmacological management has been shown to decrease symptoms, lower relapse rates, and improve quality of life [8-16]. Unfortunately, empirically supported adjunctive psychotherapies for BD can be hard to access because of barriers such as limited provider availability, clinic location, and financial burden [17,18]. These barriers stress the need for more cost-effective and accessible treatment modalities. Mental health technologies (MHTs) such as smartphone- and web-based interventions may be well-suited to increasing access to and enhancing the functionality of adjunctive psychotherapy for individuals with BD.

Over the last decade, MHTs have been developed for various mental health challenges [19-21]. Among individuals with BD, research indicates high rates of smartphone ownership [22] as well as interest and willingness to access BD-related information via technology [23]. To address access barriers and enhance treatment for individuals with BD, smartphone apps that port self-management strategies from empirically supported psychotherapies have been developed and individuals that use these apps report high levels of satisfaction [24-30]. Despite the emergence of these technology-delivered interventions, we still have limited knowledge of how individuals with BD experience these treatment formats [27].

Studies that use qualitative methods to evaluate individuals’ lived experiences while using and applying these interventions in day-to-day activities can highlight the potential benefits and disadvantages of intervention components [31]. Despite the potential of qualitative methods to elucidate factors influencing behavior change and maintenance processes [32,33], only a small number of BD MHT studies have explored how users perceive MHT use for stimulating these processes. Of the existing studies, individuals with BD reported finding MHTs usable and useful for disease management [34,35]. More specifically, they often report that mood and activity monitoring using a smartphone can help increase insight and behavior change [36]. However, current studies have not yet comprehensively examined how MHT use influences behavior change and maintenance processes related to the multiple targets and approaches proposed to underlie living well with BD.

The current paper describes a thematic analysis of in-depth exit interviews initiated immediately after participants completed a field trial for LiveWell, a smartphone-based self-management intervention for individuals with BD. The analysis presented here focuses on how individuals with BD perceive and utilize this smartphone-based intervention for health behavior change and maintenance.

## Methods

### Participants

The study was reviewed and approved by the Northwestern University Institutional Review Board. Participants were recruited via flyers describing the smartphone intervention and eligibility criteria. Flyers were placed at university-affiliated and private outpatient mental health practices. Eligible participants were 18 to 65 years old and had a Diagnostic and Statistical Manual of Mental Disorders-IV diagnosis of BD 1 with a minimum of two acute mood episodes within 2 years of enrollment. Individuals were excluded if they: (1) were not in current psychiatric care; (2) met criteria for a substance use disorder within the last 6 months; (3) met criteria for another psychiatric diagnosis or had symptoms for which participation in the study was either inappropriate or dangerous, including current severe suicidal ideation or a serious suicide attempt in the last 12 months; (4) were pregnant or planned to become pregnant; (5) had visual, hearing, voice, or motor impairment that would prevent completion of the study procedures or limit smartphone use; (6) were unable to speak or read English; or (7) were in a current mood episode at the baseline assessment.

Individuals who were interested in participation were encouraged to call the research team or contact the team via the study’s website. Before the initial telephone screening, participants provided informed consent for online or telephone screening. The initial telephone screening was conducted to establish a BD diagnosis using the Mini International Neuropsychiatric Interview [37]. If eligible, users completed a written consent form for study participation prior to engaging in a face-to-face interview with a study clinician (psychiatrist or psychologist). At the face-to-face interview, an abbreviated version of the Affective Disorders Evaluation and the Clinical Monitoring Form was used to confirm the diagnosis of BD Type 1 [38,39]. Individuals with a confirmed diagnosis at the clinic visit were scheduled for a baseline assessment. If these individuals were not in an episode at the baseline assessment, they were enrolled in the pilot study.

Participants were compensated for time and travel costs: (1) US $10 for travel costs and telephone assessment and (2) US $15 for each assessment, including the clinical assessment, baseline/monthly telephone assessment, exit interview, and app training. Eleven participants were enrolled in the pilot study. The participants were 21 to 62 years old (mean 36 years, SD 14), including 4 men and 7 women. The majority (n=11) were non-Hispanic White. In terms of relationship status, 3 were married/living as married, 3 were divorced, and 5 were never married. With respect to education, 5 participants indicated some college, one had a college degree, and five had education beyond college. Two participants were students, 6 were employed, 1 was unemployed, and 2 were on disability.

### Procedures

All participants were provided with a smartphone and a data plan and completed an 8-week pilot study. Participants had a face-to-face meeting with a coach who used a structured script and handouts to instruct them on the use of the app and the role of the coach [40]. Following the face-to-face meeting, participants completed six phone calls (weeks 1-4, 6, and 8) during which the coach used structured scripts to support app use adherence, development of personalized wellness plans, self-management strategy use, and communication with clinical care providers [40]. To provide feedback about the intervention’s impact on app usability, target behavior change processes, and clinical and recovery outcomes, participants completed a structured exit interview (Multimedia Appendix 1) and an exit questionnaire after completing the pilot study [19].

### Intervention Design

The LiveWell intervention aims to assist individuals with BD in using self-management strategies to reduce relapse risk and symptom burden as well as to improve quality of life. The LiveWell intervention has technological and human support components that include a smartphone app, secure server and website, and coach. The smartphone app has five components: Foundations, Toolbox, Wellness Plan, Daily Check In, and Daily Review [19,41]. The core of the intervention is the Daily Check In, which helps participants monitor behavioral targets proposed to be important for managing BD and staying well (medication adherence, sleep duration, routine, managing signs and symptoms) [10,11]. Participants use the smartphone app to check in daily (Daily Check In) and monitor these targets. An expert system (Daily Review) provides interactive, personalized real-time feedback based on their Daily Check In data [41]. Additionally, participants have access to psychoeducational content in the Foundations and Toolbox that helps them develop a personalized Wellness Plan, which addresses lifestyle skills for reducing risk, coping skills for managing signs and symptoms, and resources essential for staying well. In addition to addressing the targets monitored with the Daily Check In, the Foundations and Toolbox also discusses attending to healthy habits concerning substance use, diet, and exercise; managing stressors; and building and using support systems to stay well. The coach supports app use adherence, self-management skill use, and clinical care communication. An initial face-to-face meeting with the coach helps participants identify personalized wellness anchors for a wellness rating scale (0 balanced, –1/+1 daily hassles/uplifts, –2/+2 prodromal/residual symptoms, –3/+3 episode, –4/+4 crisis). The wellness scale is used during the Daily Check In for monitoring signs and symptoms [40]. Screenshots of the LiveWell intervention components can be found in Multimedia Appendix 2.

### Intervention Framework

**Figure 1:**
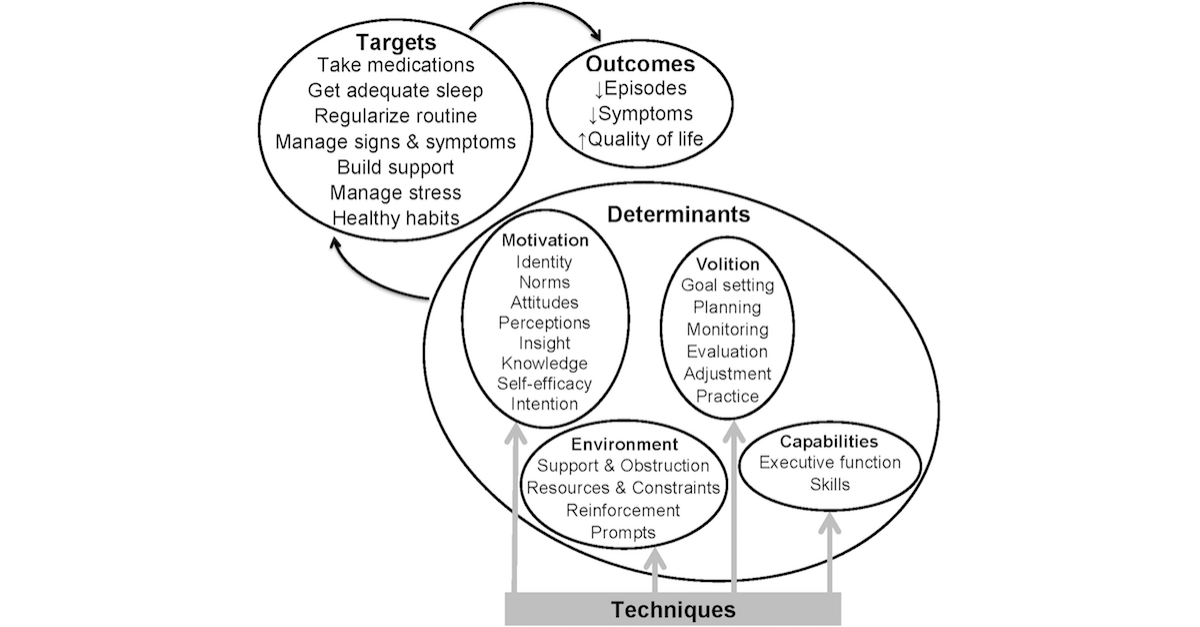
LiveWell behavior change framework.

LiveWell was designed using a behavior change framework that integrates user feedback with information from empirically supported psychotherapies for BD, health psychology behavior change theories, and chronic disease self-management models [19,41]. The framework proposes that (1) engaging in target behaviors improves clinical and recovery outcomes, (2) behavioral determinants govern enactment of target behaviors, and (3) exposure to behavior change technique content and tool use alter behavioral determinants (Figure 1). The framework integrates and organizes behavioral determinants defined in existing behavior change theories into four domains: motivational determinants involved in developing an intention to engage in a behavior, volitional determinants involved in enacting the behavior, environmental determinants, and capabilities that impact motivation and volition [32,33,42-58]. This framework guided the deductive coding performed during thematic analysis of participants’ feedback about the impact of the intervention on their behaviors and wellness.

### Analysis

The exit interviews (N=11) were transcribed verbatim and used for thematic analysis [59]. Initial codes were developed using deductive coding guided by the exit interview script (Multimedia Appendix 1) and the intervention’s behavior change framework [19]. Three researchers independently performed a preliminary round of coding during which transcripts were partitioned into excerpts (transcript lines conveying a codable unit) and exported to Microsoft Excel spreadsheets (Multimedia Appendix 3). Intervention subthemes, or emergent patterns that occurred within themes, were inductively coded and deductively grouped into larger themes and therein determinants. Coders used nominal group consensus, where they met with a moderator to discuss, clarify differences in coding, and finalize codes [60]. Following this process, the frequencies of all excerpts and codes were quantified. The decision to quantify the qualitative data was driven by the research team’s desire to clearly identify patterns as well as inconsistencies and outliers within participant responses. Quantification of qualitative findings can help researchers recognize diversity in qualitative data [61]; provide data transparency to avoid selective cherry-picking of data [62,63]; and add precision to the presentation of findings in terms of importance, frequency, or strength of findings [61,64].

A total of 210 excerpts (mean 19, SD 8 per transcript) were given 1-5 codes (110 coded once, 65 coded twice, 23 coded three times, 10 coded four times, 2 coded five times). For each excerpt, coders identified and coded whether or not participants discussed one of LiveWell’s behavioral targets (manage signs and symptoms, sleep duration, medication adherence, routine, healthy habits, build support, or none) as well as any determinants participants discussed as impacting the targets (Multimedia Appendix 3). A total of 329 codes were identified pertaining to participants’ experience of the intervention impact. To account for participants who discussed a coded element frequently, a ranking score (range from 1 to 10) was assigned at each level of coding to provide a metric of how often participants discussed a given code (code count, CC) weighted by the number of participants (participant count, PC) who discussed the code:

ranking score=10^(log[CC×PC])/max(log[CC×PC])^

Processing of the Excel spreadsheets to obtain counts and scores was completed using MATLAB (MathWorks). The scoring output including codes endorsed by only one or two participants is provided in Multimedia Appendix 3. Participant responses to the pilot study exit questionnaire (N=11), including questions assessing the intervention’s impact on outcomes, targets, and determinants (Multimedia Appendix 4), were summarized from the 7-point response scales into two categories: disagree/strongly disagree and agree/strongly agree. Results related to app usability and user-centered development, which were also assessed during the exit interview and questionnaire analysis, have been presented elsewhere [19].

## Results

### Overview of Themes and Subthemes

During the exit interviews, participants discussed targets (Table 1 and Table 2) and determinants (Table 3) that aligned with LiveWell’s behavior change framework. For some determinants, subthemes were also identified: monitoring (checking in, reflection, self-awareness), social support (bond, accountability, legitimacy; planning, goal-setting, monitoring, prompts), and knowledge (useful, basic) (Multimedia Appendix 3).

**Table 1:** Exit interview behavioral targets.

Target	Rank score (range 1-10)	Percent participants	Percent codes
Manage symptoms and signs	10.0	100.0	29.8
Routine	7.8	90.9	15.2
Sleep	7.1	81.8	11.6
Medication	6.6	63.6	13.4
Build support	6.2	81.8	9.1
Monitored^a^	4.8	63.6	5.2
Healthy habits	4.6	54.5	5.2

^a^Participants mentioned using the Daily Check In to monitor behavior but did not discuss a specific target monitored using the Daily Check In.

**Table 2:** Exit questionnaire targets.^a^

Behavior	Questions	Percent of participants	
		Disagree and strongly disagree	Agree and strongly agree
Manage signs and symptoms	My use of the app increased my ability to identify, monitor, and manage early warning signs and symptoms	0	82
Routine	My use of the app helped me maintain a more regular routine	0	55
Medication	My use of the app increased my medication adherence	18	45
Sleep	My use of the app helped me to get the recommended amount of sleep	0	45

^a^Only questions regarding targets are included here. Responses for two additional questions regarding outcomes and determinants are available in Multimedia Appendix 4.

**Table 3:** Exit interview determinants.^a^

Determinants		Rank score (range 1-10)	Percent participants	Percent codes
**Volition**		10.0	100.0	44.4
	Monitoring	10.0	100.0	34.3
	Evaluation	3.9	54.5	3.3
	Adjustment	3.7	45.5	3.3
	Planning	3.5	54.5	2.4
	Goal setting	2.0	27.3	0.9
**Environment**		8.9	100.0	31.0
	Social support	8.7	100.0	22.5
	Constraints	3.9	63.6	3.0
	Reinforcement	3.7	45.5	3.3
	Prompts	2.9	36.4	2.1
**Motivation**		8.3	100.0	24.0
	Knowledge	6.2	100.0	7.9
	Intention	4.9	54.5	7.0
	Insight	3.9	45.5	4.3
	Self-efficacy	2.7	27.3	2.1
	Attitudes	2.2	27.3	1.2
	Norms	2.2	27.3	1.2

^a^Domains and determinants are included in the table if 3 or more participants discussed them in the interviews.

### Targets

Participants discussed the Daily Check In targets (manage symptoms and signs, routine, sleep, medication use); building and using a support network (coach, family and friends, providers); and developing healthy habits around substance use, diet, and exercise. In terms of target behaviors, participants most frequently discussed the intervention’s role in assisting them with recognition and management of their early warning signs and symptoms (Table 1).

[My] strongest memory is pulling my mood back from that mild up. It was a big deal. I was really glad to have that phone in my hands when I realized that was happening.

user ID 2005, exist interview transcript start line 1118, stop line 1126; see Multimedia Appendix 3

[LiveWell] helped me realize that it’s okay to have mood variations, that’s human, which is something I’m still dealing with determining 1s and 2s, and what’s a normal variation or not.

user ID 2016, transcript lines 62-90

Participants also felt that the intervention impacted their efforts to keep a regular routine, get the right amount of sleep, take their medications, build a support system, and engage in healthy habits such as exercise (Table 1).

I definitely started trying to stay within that window of for going to bed…I started noticing when I wasn’t getting the right amount of sleep or when my schedule was very off…It helps me course correct a bit faster.

user ID 2061, transcript lines 711-732

Thinking it through helped me be aware of my behaviors and my sleep patterns especially. I’ve been really trying to work a lot with my sleep because it helps to have it there in black and white, like this is how much I slept last night, this is how much I slept every night previous.

user ID 2001, transcript lines 153-170

In responding to exit questionnaires about the intervention’s perceived utility on making changes in behaviors (Table 2), participants’ responses aligned with the thematic interviews. Most participants reported that the intervention helped increase their ability to identify, monitor, and manage early warning signs. Additionally, some participants found that the intervention helped with developing a consistent routine and optimizing sleep duration. While some participants felt that app use helped medication adherence, two participants did not find it helpful for this target behavior. However, these individuals reported 100% adherence to their medications upon starting the intervention.

### Determinants

#### Monitoring

In terms of determinants, participants most frequently discussed how monitoring their behaviors provided an opportunity to identify and make progress toward their behavioral target goals (Table 3). They pointed out that the Daily Check In was especially helpful for monitoring symptoms (Multimedia Appendix 3).

The Daily Check In [worked best for me]. Knowing that I was being monitored. That I was gonna get help or recommendations on what to do. That was great. Knowing that I was really tracking what was going on and becoming more aware of what to look for.

user ID 2041, transcript lines 787-757

In particular, participants expressed that monitoring helped with managing early warning signs (Multimedia Appendix 3).

I noticed when I was having a mild up-phase. I don’t think I would’ve noticed it without the personalized anchors that’s something that I tend to have less insight about. I’m like… I’ve had a couple of plus two days and this, this and this is happening… It was nice to look at [my Wellness Plan] and say okay I have some things that I can do to try to bring this down and if it doesn’t go down I know that I need to make a phone call.

user ID 2005, transcript lines 1104-1116

Additionally, daily monitoring enabled some participants to make plans involving their supports to improve their target behaviors such as sleep:

If I have 2 or 3 nights with less than 6 hours of sleep, something is gonna happen so I make sure my husband is the person who takes care of the kids that night and I’ll sleep in the guest bedroom.

user ID 2086, transcript lines 190-207

#### Support

After monitoring, the determinant that participants discussed most frequently as being important to behavior change was social support from coaches, family, friends, and providers (Table 3). With regard to coaching interactions, participants brought up components of the supportive accountability model, which argues that human support increases adherence through accountability to a coach who is deemed as trustworthy, benevolent, and having expertise [65]. Specifically, participants reported that they liked, trusted, or respected their coaches (bond). They also acknowledged that the coach helped to keep them responsible when they were unable to meet their mutually agreed upon goals (accountability): “I like the idea of working with the coach…Just having someone to check in with about it and kind of also be accountable to it” (user ID 2065, transcript lines 555-559).

Additionally, participants discussed the coaches’ influence due to their perceived expertise on BD-related topics such as the target behaviors (Multimedia Appendix 3): “You just felt like somebody [coach] was listening and monitoring what was going on in your life and helping you figure out if you’re going too far this way or too far that way” (user ID 2041, transcript lines 672-685).

Participants also shared that working with a coach helped them carry out volitional processes such as goal-setting, monitoring of signs and symptoms, and using prompts to work toward achieving target behaviors such as medication adherence: “My conversations with [my coach] were a little more helpful in terms of figuring out exactly what to do in terms of keeping a routine and taking my meds at the right time” (user ID 2016, transcript lines 152-160).

Similarly, participants cited components of supportive accountability (legitimacy and accountability) inspired by the involvement of their providers (Multimedia Appendix 3). For example, some participants felt comforted by the idea that their providers had access to their Daily Check In data.

[My therapist] looked at [my clinical summary] a couple times and found it useful. I see her every other week. So she referred back to it the first time…She mentioned something like “Yeah I saw this has been your pattern” and I was like “What? Oh yeah, that’s right…” It actually was kind of—can I say comforting?…There was a sense of...I don’t know the word, but just that she’s looking at it as well.

user ID 2065, transcript lines 591-633

They also noted that the intervention provided a means to share information with family members about BD and engage their support network to assist them with volitional processes such as planning and monitoring: “There was a lot of good information in there...to be more reflective of what’s going on… and to involve people more directly, specifically my daughter” (user ID 2066, transcript lines 84-92).

#### Knowledge

The third most discussed determinant was knowledge (Table 3). Most participants found the knowledge offered about BD useful and emphasized that sharing this information with friends and family was particularly beneficial.

The recommendations...[are] really good stuff to know and things I could share with my family and support people, so they know what to look out for or what I’m looking out for.

user ID 2041, transcript lines 215-234

Foundations are good for people who are maybe newer to the disease or if I were to share that with friends and family.

user ID 2086, transcript lines 166-181

However, some participants felt that the content was a review of familiar information and wanted more advanced materials*:* “[The foundations] weren’t totally new to me, because I’ve done a lot of DBT [dialectical behavioral therapy]...I’m someone who’s been through a lot of therapy” (user ID 2061, transcript lines 92-103).

#### Motivation

In addition to knowledge, participants also discussed other motivational determinants, including intention, self-efficacy, insight, attitudes, and norms (Table 3). Participants discussed their intentions and sense of self-efficacy in developing more regular routines and better sleep habits, as well as managing symptoms and signs, and taking medications.

The thing that helped me the most was trying to stick to a routine… I needed more routine.

user ID 2063, transcript lines 6-20

I have a hard time making myself follow a routine or a structure... I’m not good at doing that.

user ID 2066, transcript lines 112-121

Participants also stated that the Daily Check In and Wellness Plan helped them develop insight by building their self-awareness about symptoms and encouraging daily reflection about their illness experience.

Personalizing the information was really helpful, like within the Wellness Plan, within triggers…again just because it made me so much more aware of myself.

user ID 2066, transcript lines 720-730

[My strongest memory was] definitely the check in and the rating of myself. That was the biggest part of the check in for me to have that time to sit down and really say like okay for the last 24 hours how was I really? I’m good now but let’s think back, or I’m not doing so well, what happened in the last 24 hours? Was it situational or was it not situational?

user ID 2086, transcript lines 109-111

Moreover, participants discussed how using the app impacted their perceptions about medications and their attitudes regarding the importance of medications and sleep duration.

Medications…were my kind of thing. Not really that I had negative beliefs or anything about medications but just why they are important, and even if they don’t feel like they are important one day, they are probably important the next day.

user ID 2016, transcript lines 105-115

Regarding identifying and managing signs and symptoms, they noted that norms about what others think and do were useful and reassuring*:* “[The Wellness Plan] kind of helped to normalize things, like, or, put things more into perspective. Like if, you know, this is what the standard you know” (user ID 2063, transcript lines 183-214).

#### Volition

In addition to monitoring, participants also discussed other volitional determinants, including evaluation, adjustment, planning, and goal-setting (Table 3). Participants that engaged in evaluation also discussed how this process prompted them to adjust their behaviors to improve their overall wellness*:*

On the few times that I was having kind of some mild depression symptoms “OK you gotta dial it up” and when I was having a small bout of hypomanic symptoms “OK…dial it down don’t talk so much, slow down.”

user ID 2005, transcript lines 116-123

Similarly, some participants noted that the Daily Check In encouraged them to evaluate patterns in their behavior and whether or not these patterns aligned with their behavioral goals.

The daily check in, you have that moment of looking back and seeing what happened. When I dipped down to that 2, I realized that I was going down a path. I had my early warning signs...I went to the wellness plan and did look it over.

user ID 2086, transcript lines 219-237

#### Environment

In addition to social support, participants discussed other environmental determinants, including constraints, reinforcement, and prompts (Table 3). Participants acknowledged that their physical environment such as a new job or varying school schedules constrained their ability to make changes in their target behaviors such as routine: “It’s hard being a student and having a regular routine” (user ID 2016, transcript lines 597-613).

Participants also stated that wanting to obtain high percentage scores on the daily review feedback bar charts reinforced their efforts to take their medications and get the right amount of sleep: “I had a hard time remembering to take my medications and being motivated once I did forget to take them…with [LiveWell] you could at least say 100% every day on medication so that really helped” (user ID 2041, transcript lines 5-13).

Despite occasional difficulties with their surrounding environments, participants reported that LiveWell helped to identify physical stimuli that helped remind them to engage in a behavior, such as taking medications: “LiveWell was a reminder to take my meds. If I wasn’t going to bed, I would remember to put them close to my bed” (user ID 2016, transcript lines 638-649).

## Discussion

A person-based approach was used to explore participants’ experience of a smartphone-based self-management intervention for BD. Participants’ accounts highlighted how they perceived the intervention impacting their efforts to stay well. Deductive thematic analysis of participants’ experiences identified behavioral targets and determinants that aligned with LiveWell’s behavior change framework and several subthemes also emerged from inductive analysis.

In terms of behavioral targets, participants most frequently discussed the importance of learning about and making an effort to manage signs and symptoms, suggesting that this target’s inclusion was highly valued. Most participants also expressed that keeping a regular routine, getting the right amount of sleep, taking medications as prescribed, and engaging in healthy habits (eg, proper diet and exercise) were target behaviors they felt were necessary to address. However, some participants discussed difficulties managing these target behaviors, especially balancing the maintenance of a regular routine with environmental constraints. Furthermore, two participants, who started the intervention reporting 100% adherence with their psychiatric medication use, indicated that the intervention did not help with medication adherence. Their feedback highlights the importance of recognizing that not all targets may be applicable or relevant to all participants. Thus, addressing baseline target behavior may be useful in identifying whether or not participants need support for behavior change or maintenance concerning a specific target.

Among behavioral determinants, participants felt that monitoring played a significant role in staying well. In particular, participants felt that regular monitoring enhanced their ability to identify and manage early warning signs and symptoms. In addition, about half of the participants discussed how monitoring helped them develop a regular routine, optimize sleep duration, and adhere to medication regimes. This feedback is consistent with existing smartphone intervention studies in which individuals with BD indicated that mood and activity monitoring helped to identify the relationship between mood states, sleep, exercise, and changes in behavior [36,66]. Data from empirically supported psychotherapies for BD indicate that the ability to distinguish between early warning signs and transitioning into an episode improves clinical outcomes [67,68]. This finding suggests that monitoring using a smartphone app may lead to improved clinical outcomes for individuals with BD.

Participants also expressed that monitoring using the Daily Check In led to increased reflection and awareness that helped them manage signs and symptoms and other target behaviors. This report from participants suggests that monitoring helps individuals build insight, including awareness of having BD, the presence of symptoms and their consequences, and the need for treatment. Enhanced awareness and reflection due to monitoring may have important implications for improved outcomes [69]. Higher levels of insight about BD such as better awareness of the illness, particularly awareness of the need for treatment, is associated with better medication adherence [70], higher self-reported quality of life [71], and increased potential to slow the progression of symptoms into a full-blown mood episode [72]. Participants’ discussion of the impact of monitoring on aspects of insight such as self-awareness of signs and symptoms also reveals that determinants (ie, monitoring and insight) may interact with one another in addition to impacting targets. This interaction of determinants is consistent with chronic disease self-management models, which consider behavior change and maintenance processes as involving a continuous and reciprocal system in which multiple wellness outcomes, target behaviors, and behavioral determinants interact continuously and reciprocally to impact health behavior change [73,74].

In addition to monitoring, participants frequently described social support as critical to their efforts to stay well. Participants underscored bond, accountability, and legitimacy as crucial components of the coaches’ influence in motivating them to use the app and self-management strategies to achieve their target goals. This finding aligns with literature suggesting that the inclusion of human support helps make interventions more personally relevant and may improve engagement and decrease attrition [19,38,40,42]. Participants also expressed that coaches helped assist them with volitional determinants such as goal-setting and monitoring. In addition, participants highlighted that support from family, friends, and health care professionals was valuable for making plans and monitoring target behaviors. This feedback is consistent with previous research indicating that a lack of social support can hinder self-management and that calling on trusted individuals for assistance is essential for chronic disease self-management [75-78]. Participants’ discussion of the value of obtaining assistance from family and friends and working with their providers suggests that incorporating additional content and tools to aid participants in building support would be valued, utilized, and may improve intervention outcomes. Although the intervention contained content about building and seeking support in BD self-management, this was not a primary target behavior. The emphasis that participants placed on the important role of social supports in managing behavioral targets and staying well suggests that increasing intervention content to assist individuals in building and seeking support may be an effective means to improve self-management interventions for BD and other mental health conditions.

The significance of monitoring and social support as valuable determinants may offer insight into how to strengthen the role of these determinants in future MHTs. First, monitoring has been discussed widely as an essential component of managing wellness for BD in MHTs and traditional face-to-face therapy settings [11,19,66,79,80]. As evidenced by the targets the participants discussed in our qualitative interviews, monitoring can apply to multiple behavioral targets. Given that the BD symptom experience at the individual level varies, MHT users may want to emphasize or focus on different behavioral targets for monitoring purposes. For instance, if an individual reports self-efficacy in medication adherence but difficulty with managing sleep duration, providing said individual with an option to opt out of medication adherence or adding a more relevant behavioral target may strengthen the user’s personal connection and engagement with the MHT. Participants’ identification of social support as a meaningful determinant also has important implications for how and in what capacity to involve social elements within MHTs. LiveWell did not include an opportunity for participants to engage in peer-to-peer discussion and exchange ideas. However, previous MHTs that have integrated a social component have demonstrated that the connection and support participants generate provide positive reinforcement and encouragement to engage in target behaviors [81,82]. Our research team sought to address feedback about the value of social support by enhancing the coach assistance that participants received throughout the study [40]. Future MHTs may consider integrating social support from family, friends, and providers more readily into the intervention.

One limitation of this study is that the interviews were conducted using an interview guide primarily focused on feasibility, usability, and satisfaction. Due to this interview guide format, it is possible that these qualitative findings do not represent the breadth of responses that may have been discovered had the research team asked directly about behavior change or had used more broadly open questions about the intervention’s impact on day-to-day life and wellness. However, despite the lack of direct questioning about behavior change, participants still spoke in-depth about their experiences integrating the intervention in their behavior change processes. Another limitation is that the research team mainly used a deductive coding approach utilizing the intervention’s underlying behavior change framework, which integrates information from empirically supported psychotherapies for BD [5,6,9,12-14,83-85], health psychology behavior change theories [32,33,42-58], and chronic disease self-management models [73,86-91]. This approach may have impacted the ability to identify novel themes from participants’ responses. Nevertheless, the researchers identified several inductive subthemes such as reflection, awareness, and insight in developing their codebook. In addition, quantifying qualitative information based on themes may reduce the rich interpretation of data expressed during participant interviews [92]. Quantifying the frequency of discussion may also not capture whether participant comments are positive or negative. Finally, because of the study’s small and limited sample size, the results are likely not representative of all people who may utilize a smartphone self-management intervention for BD. This limits inferences that can be drawn about the prevalence of these findings beyond the current sample.

Utilizing qualitative approaches to understand how participants perceive the impact of technologies on their behavior change and maintenance processes provides an opportunity to understand how these technologies are integrated into their daily lives. As a result, qualitative approaches may highlight how MHTs can be developed to better meet participants’ needs. In this study, participants discussed the importance of several behavioral targets and determinants that the intervention aimed to address, suggesting that the intervention framework and design aligned with participants’ needs and interests. However, participants also emphasized the importance of gaining support from family and friends, even though support was not emphasized or extensively developed as a target in the intervention content and tools. Using person-based development approaches to move beyond examining usability to comprehensively examine how participants perceive MHTs impacting their behavior change and maintenance efforts may thus provide new ideas about how to design and improve these technologies.

## Appendix

Multimedia Appendix 1 LiveWell pilot study exit interview script.

Multimedia Appendix 2 LiveWell app screenshots.

Multimedia Appendix 3 LiveWell pilot study exit interview data.

Multimedia Appendix 4 LiveWell pilot study exit questionnaire data.

## Abbreviations

BD: bipolar disorder
CC: code count
MHT: mental health technology
PC: participant count
