# Supplementary material for: A Smartphone-Based Self-Management Intervention for Individuals with Bipolar Disorder (LiveWell): Qualitative Study on User Experiences of the Behavior Change Process
Source: JMIR Ment Health. 2021 Nov 22;8(11):e32306. doi: 10.2196/32306 (PMC8663488; doi:10.2196/32306)

LiveWell Application Homepage

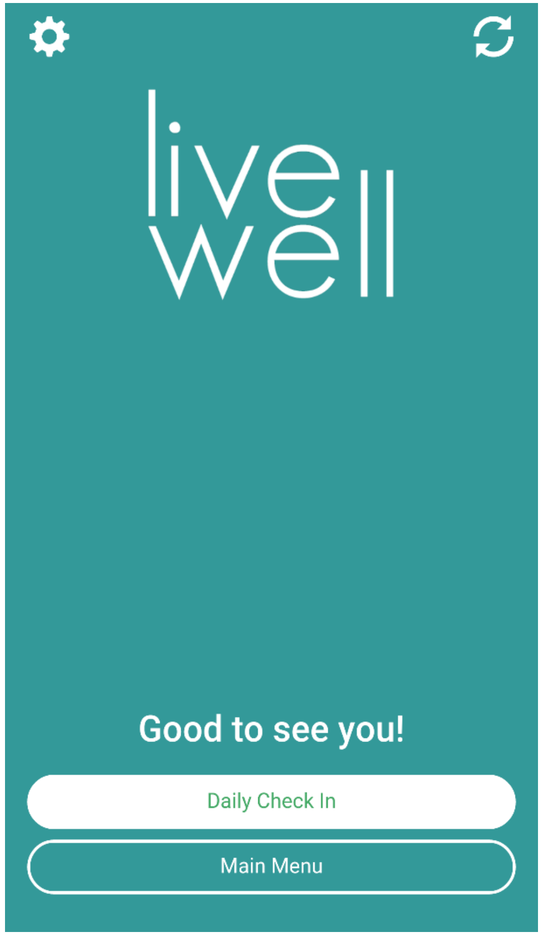

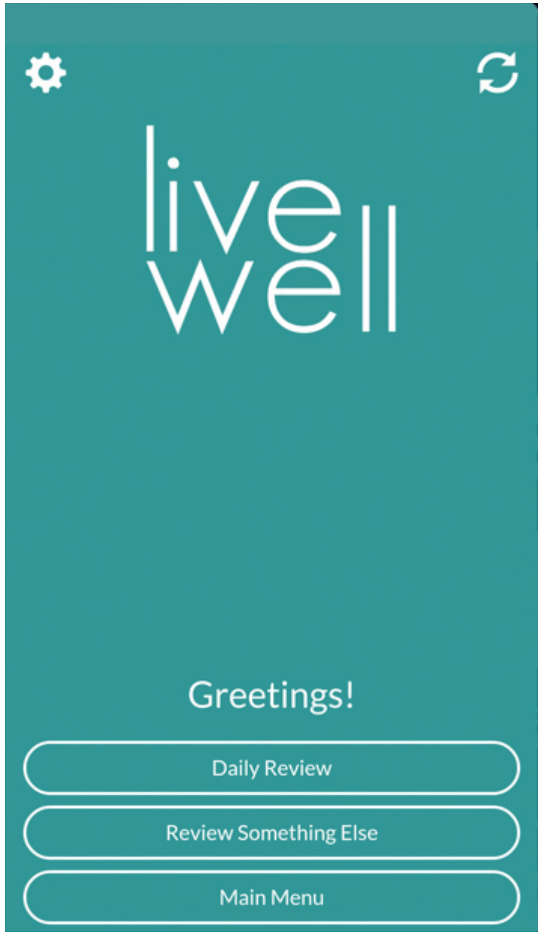

Foundations & Toolbox

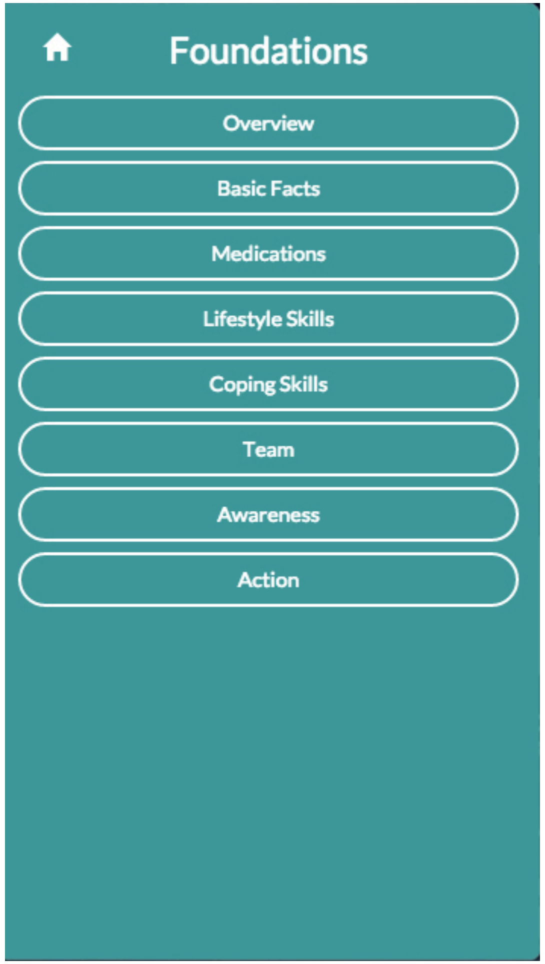

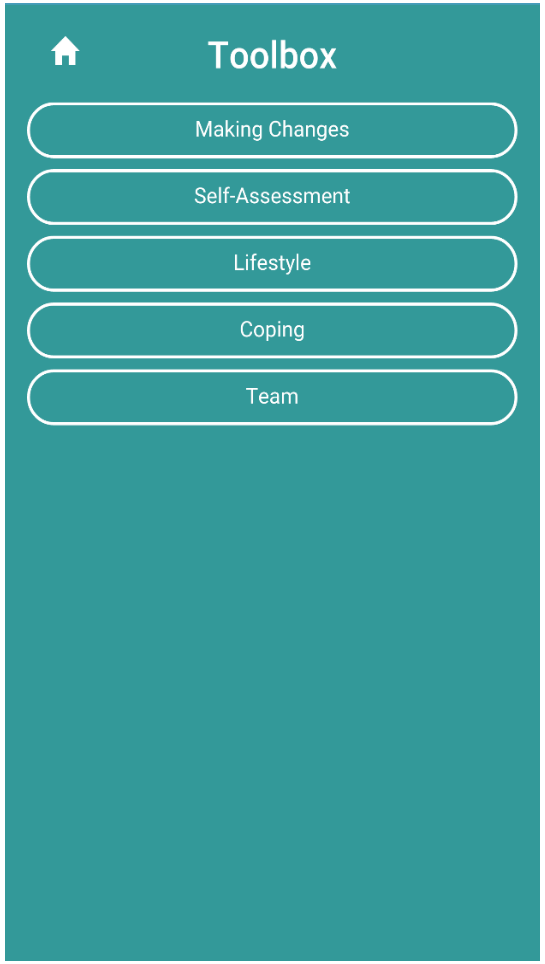

Wellness Plan

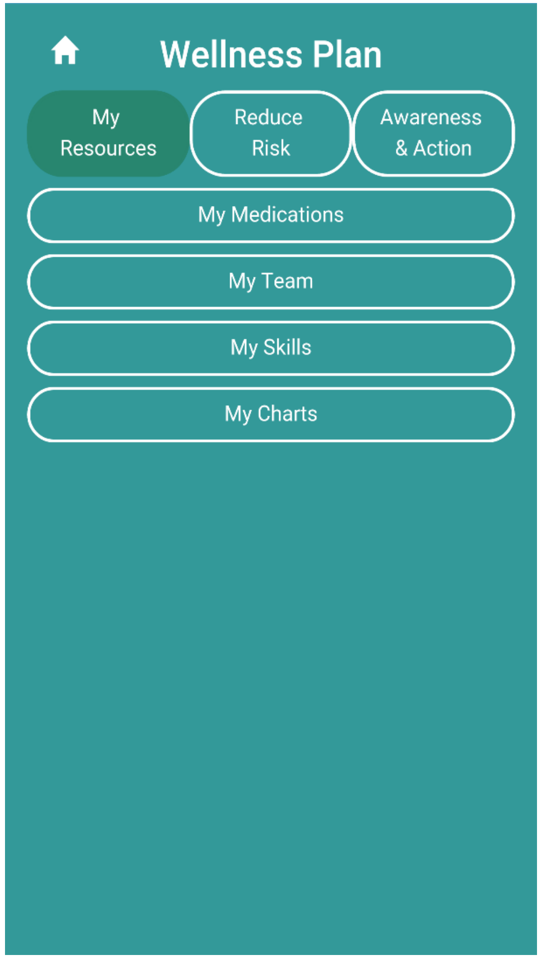

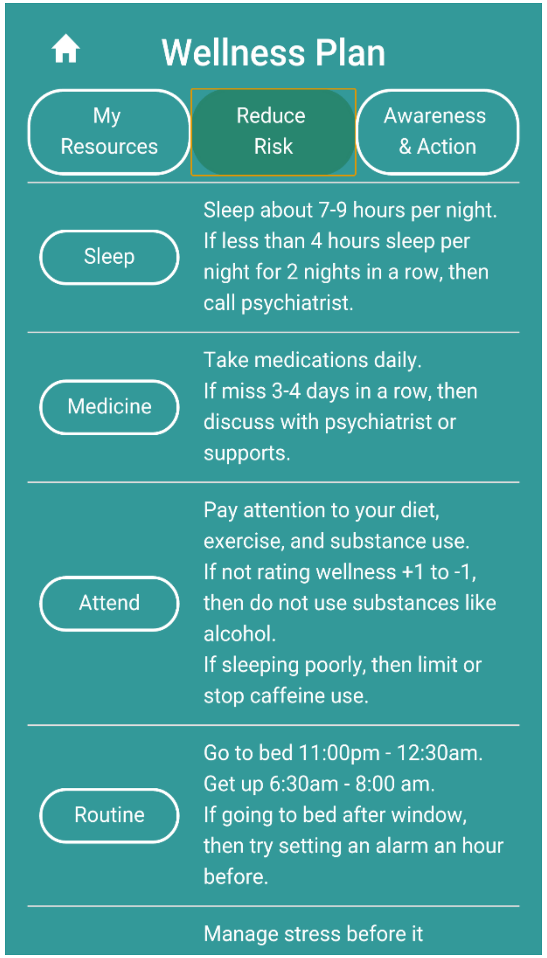

Wellness Plan

Wellness Plan

My Resources | Reduce Risk | Awareness & Action

Plan | Anchors | Definition

+4	**Severe Up**	Poor judgement. Dangerous behaviors.<br>Not sleeping.<br>Hallucinations/delusions.
+3	**Moderate Up**	Many symptoms day to day.<br>Manic episode probably happening.<br>Difficult to maintain activities/routine.
+2	**Mild Up**	Some symptoms, early warning signs.<br>Manic episode may be coming.<br>Can still maintain activities/routine.
+1	**Slight Up**	Response recent/upcoming good event.<br>Likely normal variation in wellness.<br>Understandable and manageable.

Wellness Plan

My Resources | Reduce Risk | Awareness & Action

Plan | Anchors | Definition

+4	**Severe Up**	Call psychiatrist or 911.<br>Go to the nearest emergency room.
+3	**Moderate Up**	Work more closely with psychiatrist.<br>If not improving, get more intensive treatment.
+2	**Mild Up**	Manage symptoms using coping skills.<br>Contact your supports.<br>If not improving, contact psychiatrist.
+1	**Slight Up**	Manage triggers using lifestyle skills.
0	**Balanced**	Maintain a healthy lifestyle.<br>Build skills.
-1	**Slight Down**	Manage triggers using lifestyle skills.

Daily Check In & Daily Review

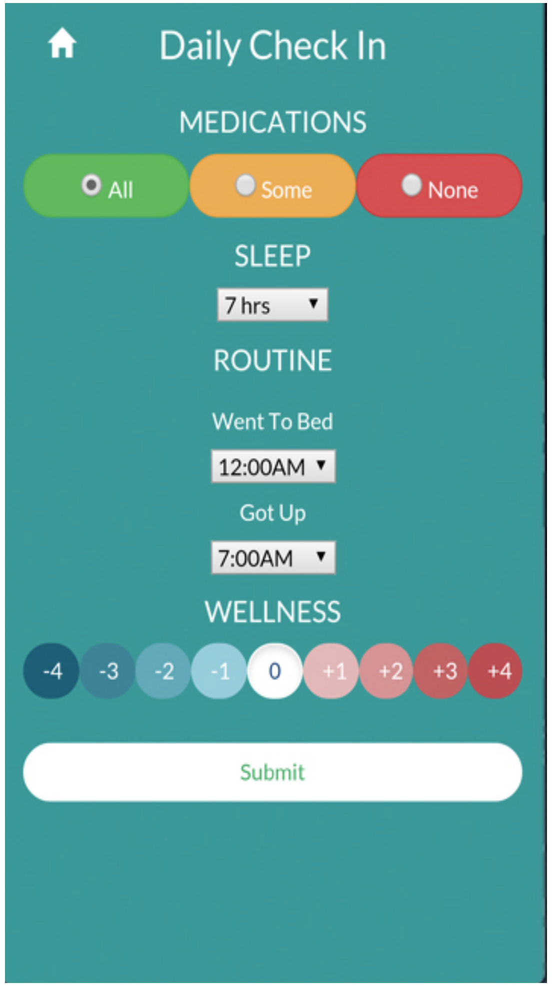

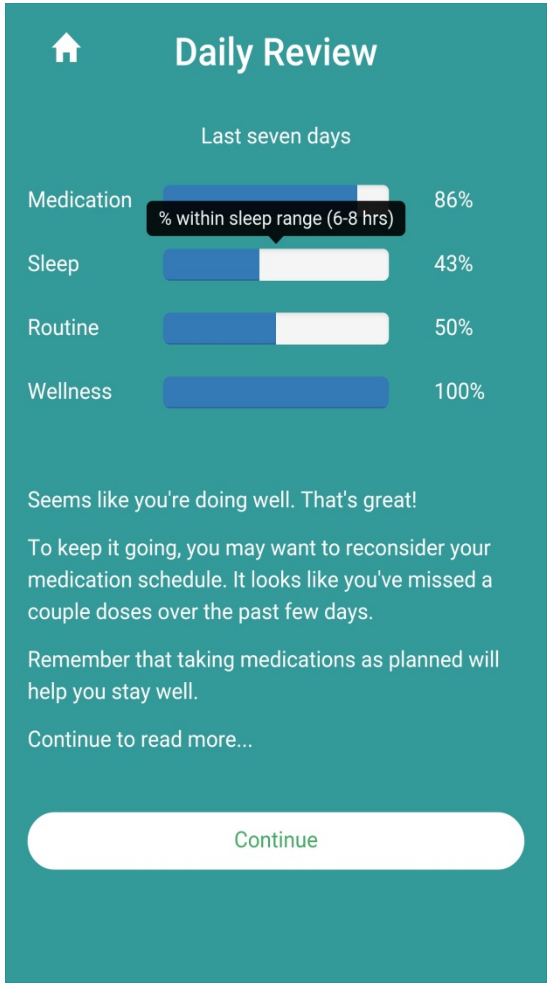

Daily Review - Psychoeducation

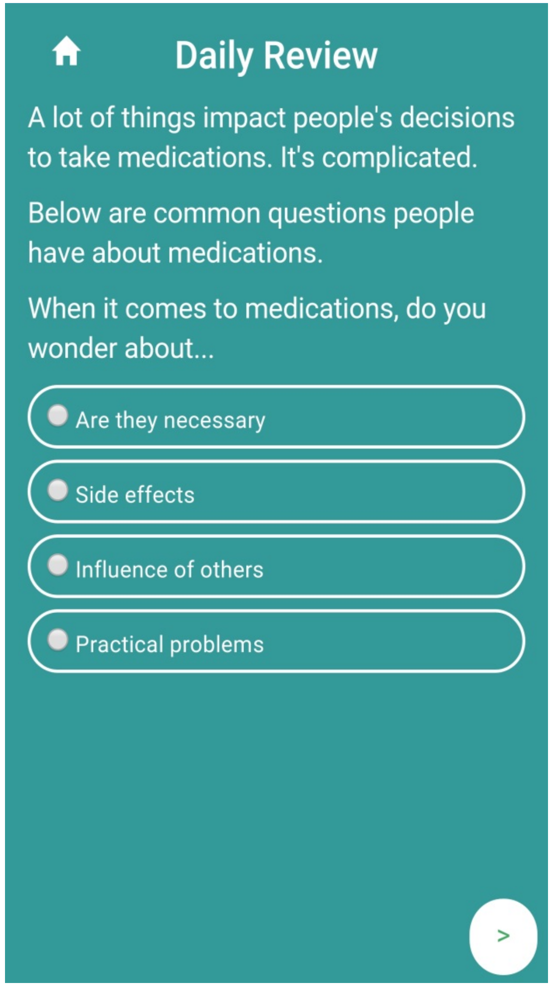

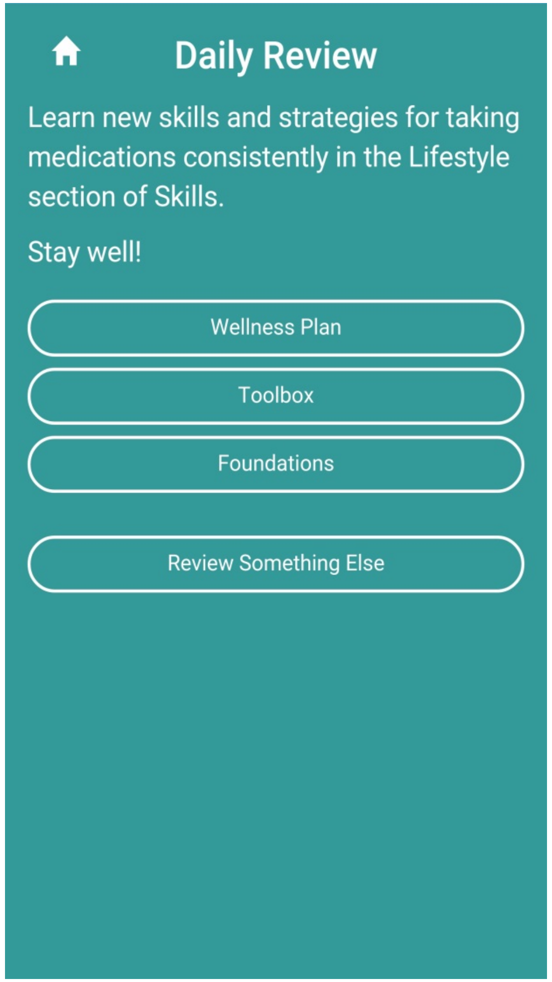

Daily Review – My Charts

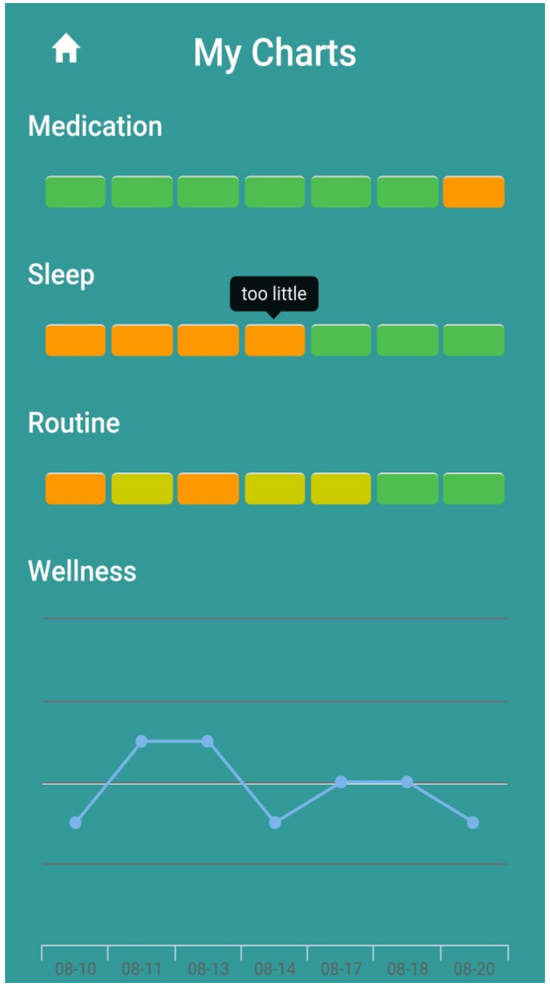

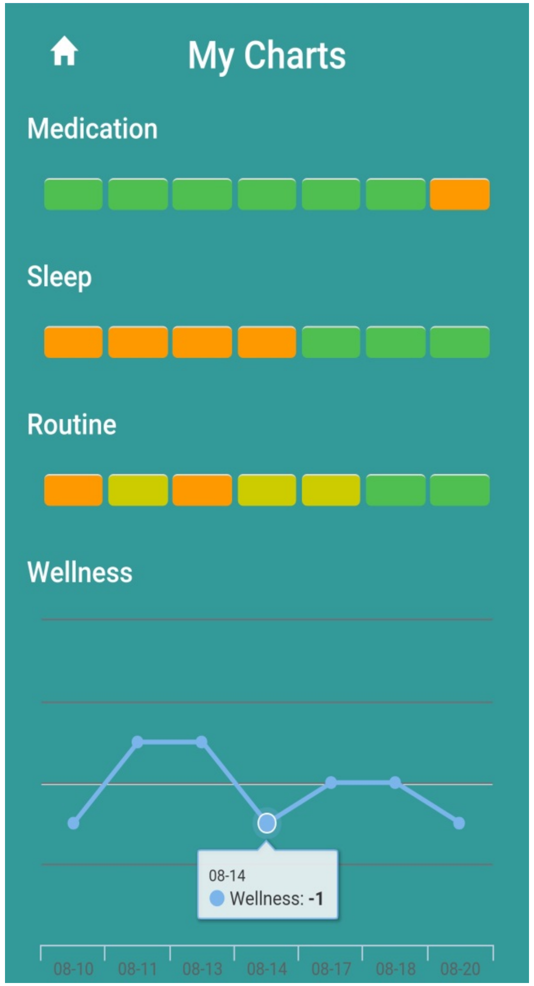

Supplement: Multimedia Appendix 2 [file mental_v8i11e32306_app2.pdf]
